# Maintained high sustained serum malondialdehyde levels after severe brain trauma injury in non-survivor patients

**DOI:** 10.1186/s13104-019-4828-5

**Published:** 2019-12-03

**Authors:** Leonardo Lorente, María M. Martín, Pedro Abreu-González, Luis Ramos, Juan J. Cáceres, Mónica Argueso, Jordi Solé-Violán, Alejandro Jiménez, Victor García-Marín

**Affiliations:** 1 Intensive Care Unit, Hospital Universitario de Canarias, Ofra, s/n. La Laguna, 38320 Santa Cruz de Tenerife, Spain; 2 Intensive Care Unit, Hospital Universitario Nuestra Señora de Candelaria, Crta del Rosario s/n, 38010 Santa Cruz de Tenerife, Spain; 3 Deparment of Phisiology, Faculty of Medicine, University of the La Laguna, Santa Cruz de Tenerife, Spain; 4 Intensive Care Unit, Hospital General La Palma, Buenavista de Arriba s/n, Breña Alta, 38713 La Palma, Spain; 5 Intensive Care Unit, Hospital Insular, Plaza Dr. Pasteur s/n, 35016 Las Palmas de Gran Canaria, Spain; 6 Intensive Care Unit, Hospital Clínico Universitario de Valencia, Avda Blasco Ibáñez nº17-19, 46004 Valencia, Spain; 7 Intensive Care Unit, Hospital Universitario Dr. Negrín, CIBERES, Barranco de la Ballena s/n, 35010 Las Palmas de Gran Canaria, Spain; 8 Research Unit, Hospital Universitario de Canarias, Ofra, s/n. La Laguna, 38320 Santa Cruz de Tenerife, Spain; 9 Deparment of Neurosurgery Hospital, Universitario de Canarias, Ofra, s/n. La Laguna, 38320 Santa Cruz de Tenerife, Spain

**Keywords:** Malondialdehyde, Brain trauma, Patients, Mortality, Injury

## Abstract

**Objective:**

Higher blood malondialdehyde (biomarker of lipid peroxidation) levels in the first hours of traumatic brain injury (TBI) have been found in patients with a worst prognosis. The objective of this study was to determine whether serum malondialdehyde levels during the first week of severe TBI could be used as mortality biomarkers. This was a multicenter, prospective and observational study performed in six Spanish Intensive Care Units. We included patients with severe TBI (defined as Glasgow Coma Scale < 9), and with Injury Severity Score in non-cranial aspects < 9. We determined serum malondialdehyde concentrations at days 1, 4 and 8 of TBI. We stablished 30-day mortality as the end-point study.

**Results:**

We found that serum malondialdehyde concentrations at days 1 (p < 0.001), 4 (p < 0.001), and 8 (p < 0.001) of TBI were higher in non-survivor (n = 34) than in survivor (n = 90) patients. We found an area under curve of serum malondialdehyde concentrations at days 1, 4, and 8 of TBI to predict 30-day mortality of 77% (p < 0.001), 87% (p < 0.001) and 84% (p < 0.001) respectively. Thus, the new and most relevant findings of our study were serum malondialdehyde levels during the first week of TBI could be used as mortality biomarkers.

## Introduction

Traumatic brain injury (TBI) leads to many deaths, and also to many disabilities and consumption of resources [1]. In TBI could appears a secondary brain injury during the following hours or days after TBI due to the neuroinflammatory response and the generation of free radicals [2–5].

Under physiologic conditions, the production of reactive oxygen species (ROS) in brain tissue is balanced by the action of antioxidant agents. However, in TBI appears an increase in the production of ROS and this leads to lipid peroxidation. The degradation of cellular membrane phospholipids during this lipid peroxidation leads to the production of Malondialdehyde, which is an end-product of lipid peroxidation [6, 7], comes to extracellular space and afterwards to the blood; and its determination has been used to estimate lipid oxidation [6, 7].

Previous studies have reported higher blood levels of malondialdehyde in TBI patients than in controls subjects [8–15]. In addition, higher blood malondialdehyde levels in the first hours of TBI in patients with a worst prognosis have been found [13–16]. Thus, the objective of this study was to determine serum malondialdehyde levels during the first week of a severe TBI and to analyze whether those levels during the first week are difference between survivor and non-survivor patients, and whether could be used as biomarkers of mortality.

## Main text

### Methods

#### Design and subjects

We performed this prospective and observational in 6 Intensive Care Units of Spain. The Institutional Ethic Review Board of each hospital approved the study: Hospital Universitario Nuestra Señora de Candelaria of Santa Cruz de Tenerife, Hospital Clínico Universitario of Valencia, Hospital General of La Palma, Hospital Insular of Las Palmas de Gran Canaria, Hospital Universitario de Canarias of La Laguna, and Hospital Universitario Dr. Negrín of Las Palmas de Gran Canaria. Besides, legal guardians of patients signed the informed consent form to study participation.

Patients with severe TBI, defined as Glasgow Coma Scale (GCS) [17] < 9 points, and with Injury Severity Score (ISS) [18] in non-cranial aspects < 9 points were included. We excluded patients with malignant or inflammatory disease, age < 18 years, pregnancy or comfort measures only.

In a previous study by our team were determined serum malondialdehyde concentrations in the day of TBI in some of those patients [13]. In our current research, we determine serum concentrations of malondialdehyde in the day 1, 4 and 8 of TBI in 118 patients.

#### Clinical and demographic variables

We recollected Acute Physiology and Chronic Health Evaluation II (APACHE II) score [19] and brain lesions by computer tomography (CT) Marshall classification [20], and other clinical and demographic variables as in our previous study [13]. We established 30-day mortality as the end-point of the study.

#### Collection of blood samples and determination of serum malondialdehyde levels

Collection of blood samples were obtained on days 1, 4 and 8 of TBI and were processed as in our previous study [13]. Determination of serum malondialdehyde levels was carried out with thiobarbituric acid-reactive substance (TBARS) method of Kikugawa et al. [21] with some modifications as in our previous study [13].

#### Statistical methods

We carried out statistical analyses similarly as in our previous study [13], using Wilcoxon–Mann–Whitney test and Chi square test to compare variables between patient groups, multiple logistic regression analysis to determine the association between serum malondialdehyde levels and 30 day-mortality, and Spearman’ coefficient to determine the association between continuous variables. In addition, to determine the capacity of serum malondialdehyde levels at day 1, 4 and 8 of TBI for 30-day mortality, receiver operating characteristic (ROC) analyses were performed.

### Results

**Fig. 1:**
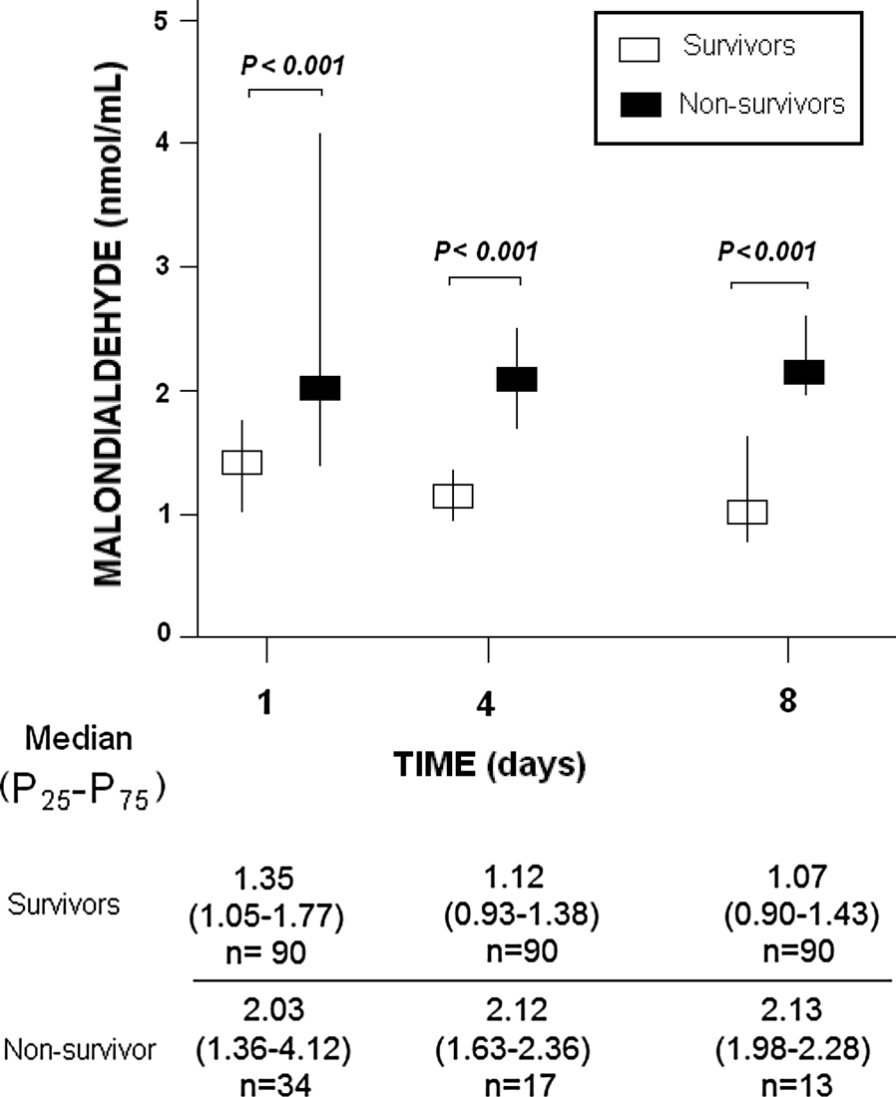
Serum malondialdehyde levels at day 1, 4 and 8 of trauma brain injury in 30-day survivor and non-survivor patients

In Table 1 appears demographic and clinical variables of non-surviving (n = 34) and surviving patients (n = 90). Non-surviving TBI patients showed higher female rate, APACHE-II score and age, and lower GCS than survivors. Besides, CT findings were different in surviving and non-surviving patients. We found that non-survivor in respect to survivor patients showed higher serum malondialdehyde concentrations at days 1 (p < 0.001), 4 (p < 0.001), and 8 (p < 0.001) of TBI (Fig. 1).

**Table 1:** Clinical and biochemical characteristics of 30-day survivor and non-survivor trauma brain injury patients

	Non-survivors (n = 34)	Survivors (n = 90)	p Value
Computer tomography classification—n (%)			0.01
Type 1	0	0	
Type 2	5 (14.7)	25 (27.8)	
Type 3	6 (17.6)	15 (16.7)	
Type 4	9 (26.5)	13 (14.4)	
Type 5	6 (17.6)	32 (35.6)	
Type 6	8 (23.5)	5 (5.6)	
CT with high risk of death (types 3, 4, 6)—n (%)	23 (67.6)	33 (36.7)	0.002
Gender female—n (%)	13 (38.2)	15 (16.7)	0.02
Age (years)—median (p 25–75)	65 (55–75)	46 (28–62)	< 0.001
Temperature (°C)—median (p 25–75)	36.0 (35.0–37.0)	37.0 (36.0–37.3)	0.07
Creatinine (mg/dl)—median (p 25–75)	0.80 (0.70–1.10)	0.80 (0.70–1.00)	0.50
Glycemia (g/dL)—median (p 25–75)	160 (125–191)	139 (121–167)	0.11
Sodium (mEq/L)—median (p 25–75)	141 (136–147)	140 (138–143)	0.41
Bilirubin (mg/dl)—median (p 25–75)	0.70 (0.53–1.05)	0.60 (0.40–0.80)	0.06
Lactic acid (mmol/L) median (p 25–75)	2.30 (1.25–4.58)	1.75 (1.10–2.50)	0.08
PaO2 (mmHg)—median (p 25–75)	142 (97–195)	148 (110–242)	0.45
PaO2/FI0_2_ ratio—median (p 25–75)	294 (167–395)	336 (246–400)	0.11
Leukocytes—median*10^3^/mm^3^ (p 25–75)	14.9 (9.7–21.6)	13.9 (10.1–19.0)	0.47
Fibrinogen (mg/dl)—median (p 25–75)	348 (300–475)	371 (286–471)	0.70
aPTT (seconds)—median (p 25–75)	29 (25–37)	28 (25–31)	0.25
INR—median (p 25–75)	1.12 (1.03–1.48)	1.11 (1.00–1.24)	0.19
Platelets—median*10^3^/mm^3^ (p 25–75)	172 (125–232)	182 (135–238)	0.49
Hemoglobin (g/dL)—median (p 25–75)	11.9 (10.0–13.7)	11.2 (10.0–13.0)	0.73
ISS—median (ppe 25–75)	25 (25–25)	25 (25–34)	0.28
Glasgow Coma Scale score—median (p 25–75)	4 (3–7)	7 (5–8)	< 0.001
APACHE–II score—median (p 25–75)	25 (23–28)	18 (14–22)	< 0.001
ICP (mmHg)—median (p 25–75)	25 (11–30)	15 (14–20)	0.36
CPP (mmHg)—median (p 25–75)	61 (52–70)	68 (57–70)	0.60
Malondialdehyde (nmol/mL)—median (p 25–75)	2.03 (1.36–4.12)	1.35 (1.05–1.77)	< 0.001

*PaO*_*2*_ pressure of arterial oxygen/fraction inspired oxygen, *FIO*_*2*_ pressure of arterial oxygen/fraction inspired oxygen, *aPTT* activated partial thromboplastin time, *INR* international normalized ratio, *ISS* Injury Severity Score, *GCS* Glasgow Coma Scale, *APACHE II* Acute Physiology and Chronic Health Evaluation, *ICP* intracranial pressure, *CPP* cerebral perfusion pressure, *p 25–75* percentile 25th–75th

We found in ROC curve analyses an area under curve of serum malondialdehyde concentrations at days 1, 4, and 8 of TBI to predict 30-day mortality of 77% (p < 0.001), 87% (p < 0.001) and 84% (p < 0.001) respectively (Table 2).

**Table 2:** Receiver operation characteristic analysis using serum malondialdehyde levels at day 1, 4 and 8 of trauma brain injury as predictor of mortality at 30 days

	Day 1	Day 4	Day 8
Cut-off of malondialdehyde (mmol/mL)	> 1.96	> 1.83	> 1.83
AUC, 95% CI, and *p* value	0.77 (0.68–0.84) p < 0.001	0.87 (0.79–0.93) p < 0.001	0.84 (0.75–0.90) p < 0.001
Sensitivity and 95% CI	56% (38%–73%)	77% (50%–93%)	85% (55%–98%)
Specificity and 95% CI	84% (75%–91%)	92% (85%–97%)	86% (77%–92%)
Positive likelihood ratio and 95% CI	3.6 (2.0–6.3)	9.8 (4.6–21.8)	5.9 (3.4–10.2)
Negative likelihood ratio and 95% CI	0.5 (0.4–0.8)	0.4 (0.2–0.7)	0.2 (0.1–0.6)
Positive predicted value and 95% CI	58% (44%–71%)	65% (44%–81%)	46% (33%–60%)
Negative predicted value and 95% CI	84% (78%–88%)	93% (88%–96%)	98% (92%–99%)

*AUC* area under curve, *CI* confidence intervals

We found in multiple logistic regression analysis an association between serum malondialdehyde concentrations and mortality controlling for sex, CT, age and CGS (OR = 3.91; 95% CI 1.61–9.50; p = 0.003). CT findings were including in the regression analysis as CT findings with low risk of death (including CT types 2 and 5) and with high risk of death (including CT types 3, 4 and 6). That classification was based in the fact that we found a mortality rate of 16.7% (5/30) in patients with CT type 2, 28.6% (6/21) with type 3, 40.9% (9/22) with type 4, 15.8% (6/38) with type 5 and 61.5% (8/13) with type 6.

We found an association of serum melatonin levels with GCS (rho = − 0.21; p = 0.02), but not with age (rho = 0.11; p = 0.24). In addition, we found higher serum melatonin levels in female than in male (p = 0.01).

### Discussion

The new and most relevant findings of our study were that non-survivor TBI patients showed higher serum malondialdehyde levels during the first week of TBI that survivors, and that those levels during the first week of TBI could be used as mortality biomarkers.

Previously there were reported higher circulating levels of malondialdehyde in TBI patients than in controls subjects [8–15], and higher malondialdehyde levels on the first hours of TBI in patients with worst prognosis [13–16]. Higher malondialdehyde levels in erythrocytes obtained within 24 h of TBI in non-survivor than in survivor TBI patients have been found in a study by Nayak et al. [14]. Higher malondialdehyde levels in cerebrospinal fluid obtained by lumbar puncture during the first day of TBI in non-survivor than in survivor TBI patients have been found in the study by Kasprzak et al. [15]. Concentrations of malondialdehyde in blood from jugular bulb blood samples and from peripheral venous blood samples were obtained from 30 TBI patients at admission, and at 6, 12, 24 and 48 h after TBI in a study by Paolin et al. [16]; and malondialdehyde levels in jugular bulb blood samples at 6 and 12 h after TBI were higher in patients with poorer than in patients with a good 6-month neurological outcome [16]. Higher serum malondialdehyde levels on the first day of TBI obtained from a central venous catheter in non-survivor than in survivor TBI patients have been found in another study by our team [13]. Thus, the finding of our current study that serum malondialdehyde levels obtained from a central venous catheter during the first week of a severe TBI were higher in survivor than in non-survivor patients is a novel finding.

Another interesting finding of our study was the significant negative association between serum melatonin levels and GCS; thus, patients with higher clinical impairment showed higher oxidative damage.

The antioxidant administration of different agents, such as melatonin [24, 25] or memantine [26], has reduced the increase of brain tissue malondialdehyde levels in animal models of TBI. In addition, the administration of different antioxidant, such as alpha-phenyl-tert-butyl-nitrone or melatonin, has reduced the neurological deficit in animal models of TBI [27]. In a randomized clinical trial of TBI patients of small sample size (18 patients with and 14 without the administration of amantadine), the patient group receiving amantadine showed lower serum malondialdehyde levels and mortality rate (6% vs 43%) [28].

We believed that those higher serum malondialdehyde levels during the first week after TBI in non-survivor patients found in our study reflects a higher ROS production and a higher lipid peroxidation in non-survivor TBI patients. That higher production of ROS production in non-surviving TBI patients could play a rol on microvascular regulation loss and vasogenic edema formation, cellular dysfunction, and patient death [22, 23]. Thus, the administration of antioxidant agents could be a therapeutic way to explore in those patients.

### Conclusions

The new and most relevant findings of our study were that non-survivor TBI patients showed higher serum malondialdehyde levels during the first week of TBI that survivors, and that those levels during the first week of TBI could be used as mortality biomarkers.

## Limitations

Some limitations should be recognized in our study. First, the determination of other compounds of oxidant and antioxidant states was not made. Second, we have only determined malondialdehyde levels in blood from a central venous catheter, and not in cerebrospinal fluid and in blood from jugular bulb. However, in some studies was found a worst prognosis in TBI patients with high levels of malondialdehyde on the first hours after TBI in cerebrospinal fluid [15] and in blood from jugular bulb [16].

## Data Availability

The datasets used and/or analysed during the current study are available from the corresponding author on reasonable request.

## Abbreviations

ICU: Intensive Care Unit
PaO_2_: pressure of arterial oxygen/fraction inspired oxygen
FIO_2_: pressure of arterial oxygen/fraction inspired oxygen
INR: international normalized ratio
aPTT: activated partial thromboplastin time
ICP: intracranial pressure
CPP: cerebral perfusion pressure
ISS: Injury Severity Score
GCS: Glasgow Coma Scale
SOFA: Sepsis-related Organ Failure Assessment
APACHE: Acute Physiology and Chronic Health Evaluation
